# Community leadership is key to effective HIV service engagement for female sex workers in Africa

**DOI:** 10.1002/jia2.26425

**Published:** 2025-03-07

**Authors:** Primrose Matambanadzo, Lilian Otiso, Sibonile Kavhaza, Parinita Bhattacharjee, Frances M. Cowan

**Affiliations:** ^1^ Centre for Sexual Health and HIV and AIDS Research (CeSHHAR) Harare Zimbabwe; ^2^ LVCT Health Nairobi Kenya; ^3^ Women Against All Forms of Discrimination (WAAD) Harare Zimbabwe; ^4^ Institute of Global Public Health University of Manitoba Winnipeg Manitoba Canada; ^5^ Partners for Health and Development in Africa Nairobi Kenya; ^6^ Liverpool School of Tropical Medicine Liverpool UK

1

Although overall HIV incidence has declined across sub‐Saharan Africa since 2010, HIV incidence among female sex workers is nine times higher than among cisgender women [1]. Young women who sell sex are particularly vulnerable. Women who sell sex do so in the context of discrimination and intense stigma, exacerbated by the criminalization of sex work [2]. Despite impressive population‐level gains in treatment cascade engagement, antiretroviral therapy (ART) coverage and rates of viral suppression have remained lower among African female sex workers than in the general population [3]. Addressing female sex workers’ specific HIV prevention and treatment needs remains central to a comprehensive HIV response and remains one of UNAIDS central pillars for “ending AIDS by 2030.”

Community‐led, person‐centred prevention and treatment services that address contextually important barriers to service engagement, while considering sex workers’ heterogeneity and multiple intersecting vulnerabilities, remain essential [4]. Community empowerment approaches seek to build social cohesion, psychological and financial resilience, and facilitate sex workers’ ability to work collaboratively towards shared goals, enabling them to prioritize and address the specific challenges they face including barriers to uptake of, and engagement in, HIV services. There is compelling evidence that community empowerment of female sex workers increases the impact of programmes in Asia [5] and South America [6] where sex worker‐led programmes are estimated to have averted hundreds of thousands of HIV infections among female sex workers and the general population. Evidence of impact is building in Africa, where community‐led approaches have more recently been introduced, resulting in increased effective coverage of HIV services [7, 8].

Community empowerment is a process which takes time and resources to develop in any population, but possibly more so among sex workers who are marginalized, stigmatized and may be distrustful. It necessitates moving from providing services *for the community* to services being led and provided *by the community* [6]. For example, sex worker provision of services *for the community* might include mobilizing communities to engage with HIV services, deliver health education, distribute condom and HIV self‐test kits; whereas when *sex workers lead* the service provision, they receive funding directly to commission and monitor the quality of health services, they design and implement both health (e.g. community delivery of pre‐exposure prophylaxis (PrEP) or ART to ensure effective community coverage) and social programmes (e.g. violence mitigation or savings schemes) [9]. The UNAIDS Strategy for 2021–2025 states that 30% of key population programmes, including those for sex workers, should be community‐led by 2025. Comprehensive peer‐led services have been established, some scaled nationally, in several settings [1, 2, 3], with services led by communities to a greater or lesser extent.

Critically, the aim of providing community‐led services is not to task‐shift provision to less highly trained cadres who are paid less for their time, but to ensure that the services that are provided address community needs and priorities. UNAIDS, through their Equitable Financing Practice, is developing methods to accurately measure the cost of community provision where the community is both provider and beneficiary. The community needs to be fully part of the collection, analysis and interpretation of their own cost data in order to facilitate a process by which sex workers are adequately compensated for their provision of services. Additionally, community‐led “citizen science” or community‐led monitoring can increase the accountability of service providers, as well as help tailor strategies to specific settings and populations [10].

Sex worker programmes have largely relied on external donor rather than domestic funding in high‐burden countries. However, HIV funding has flatlined in recent years [11] and it is expected to continue to fall. Increasing domestic funding is unlikely to replace donor funding in full. Given the stigma and illegality associated with sex work, African governments may prioritize treatment for the general population rather than programmes designed for and by sex workers. In an effort to make services more sustainable (affordable for and acceptable to national governments), there is a move to channel donor funds for key population services away from non‐governmental or community‐based organization providers and to redirect these funds to governments to integrate services into the public sector [4]. However, sex worker communities have concerns about their privacy and confidentiality (particularly related to data), as well as the stigma and discrimination they may face in the public sector [12]. Importantly, models for creating meaningful partnership between the public sector and communities at scale (including but not limited to sex workers) are lacking in Africa, and evidence on how best to achieve this is needed.

Funding sex worker programmes (local and global funding) is good for the health of everyone [5, 6]. Male partners of female sex workers play a fundamental role in HIV transmission [13]. There is evidence emerging that female sex worker programmes can be leveraged to reach male sex partners [14]. Moreover, funding female sex worker programmes can contribute to a reduction of vertical transmission of HIV among sex workers.

The push for mainstreaming peer‐led services into public health services as the primary way of sustainably delivering HIV interventions threatens to undermine the gains made over the last 20 years [5, 8]. The sex worker community is not homogeneous, with sub‐populations including young women who sell sex, women practicing in different settings, all with different needs and priorities. Mainstreaming may standardize the programme in such a way that the needs and priorities of all sex workers cannot be met. Peer‐led programmes offer comprehensive programmes that address the wider social as well as health needs of sex worker communities. Additionally, sex workers are critical in supporting peers to take up prevention and treatment services, and can also play a significant role in building the capacity of health providers on the needs of sex workers, ensuring sustained quality and accountability.

Failing to keep sex workers engaged with HIV services may result in ongoing HIV transmission through sex work [15]. Modelling suggests that now is the time to increase the intensity of HIV prevention programmes for female sex workers in Africa, both to optimize impact among sex workers and to ensure cost‐effective and beneficial impact on population incidence more broadly [16]. Adequate funding for sufficiently high‐intensity, community‐led programmes should be considered as both “a moral imperative and a strategic necessity for global public health” [14]. Any transitions to mainstreaming services should be guided by the needs and recommendations of the sex worker community based on evidence they collect, and not be based purely on financial or donor considerations.

## COMPETING INTERESTS

The authors declare no competing interests.

## AUTHORS’ CONTRIBUTIONS

All authors contiruted ideas to shape this article. FMC and PM wrote a first draft of the paper and all authors contributed to subsequent drafts.
